# Single SNP- and pathway-based genome-wide association studies for beak deformity in chickens using high-density 600K SNP arrays

**DOI:** 10.1186/s12864-018-4882-8

**Published:** 2018-06-28

**Authors:** Hao Bai, Yanyan Sun, Nian Liu, Fuguang Xue, Yunlei Li, Songshan Xu, Jianhua Ye, Lei Zhang, Yu Chen, Jilan Chen

**Affiliations:** 1 Key Laboratory of Animal (Poultry) Genetics Breeding and Reproduction, Ministry of Agriculture, Institute of Animal Science, Chinese Academy of Agricultural Sciences, Beijing, 100193 China; 2 CapitalBio Corporation, Beijing, 102206 China; 3 Beijing General Station of Animal Husbandry Service, Beijing, 102200 China

**Keywords:** Beijing-you chickens, Beak deformity, SNP, Pathway, GWAS

## Abstract

**Background:**

Beak deformity, typically expressed as the crossing of upper and lower mandibles, is found in several indigenous chicken breeds, including the Beijing-You chickens studied here. Beak deformity severely impairs the birds’ growth and welfare. Although previous studies shed some light on the genetic regulation of this complex trait, the genetic basis of this malformation remains incompletely understood.

**Results:**

In this study, single SNP- and pathway-based genome-wide association studies (GWASs) were performed using ROADTRIPS and SNP ratio test (SRT), respectively. A total of 48 birds with deformed beaks (case) and 48 normal birds (control) were genotyped using Affymetrix 600 K HD genotyping arrays. As a result, 95 individuals and 429,539 SNPs were obtained after quality control. The *P*-value was corrected by a Bonferroni adjustment based on linkage disequilibrium pruning. The single SNP-based association study identified one associated SNP with 5% genome-wide significance and seven suggestively associated SNPs. Four high-confidence genes, *LOC421892*, *TDRD3*, *RET*, and *STMN1*, were identified as the most promising candidate genes underlying this complex trait in view of their positions, functions, and overlaps with previous studies. The pathway-based association study highlighted the association of six pathways with beak deformity, including the calcium signaling pathway.

**Conclusions:**

Potentially useful candidate genes and pathways for beak deformity were identified, which should be the subject of further functional characterization.

**Electronic supplementary material:**

The online version of this article (10.1186/s12864-018-4882-8) contains supplementary material, which is available to authorized users.

## Background

**Fig. 1:**
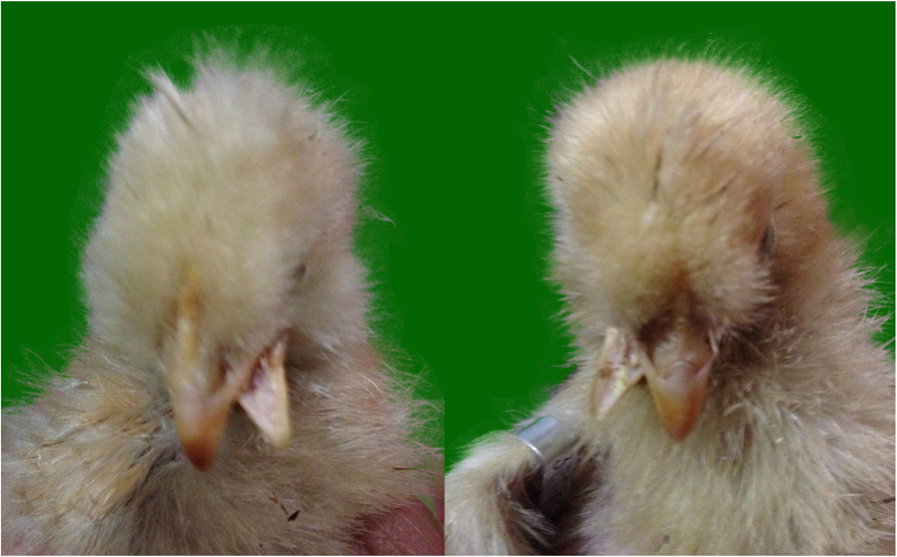
Examples of beak deformity birds of Beijing-You chickens used in the study

**Fig. 2:**
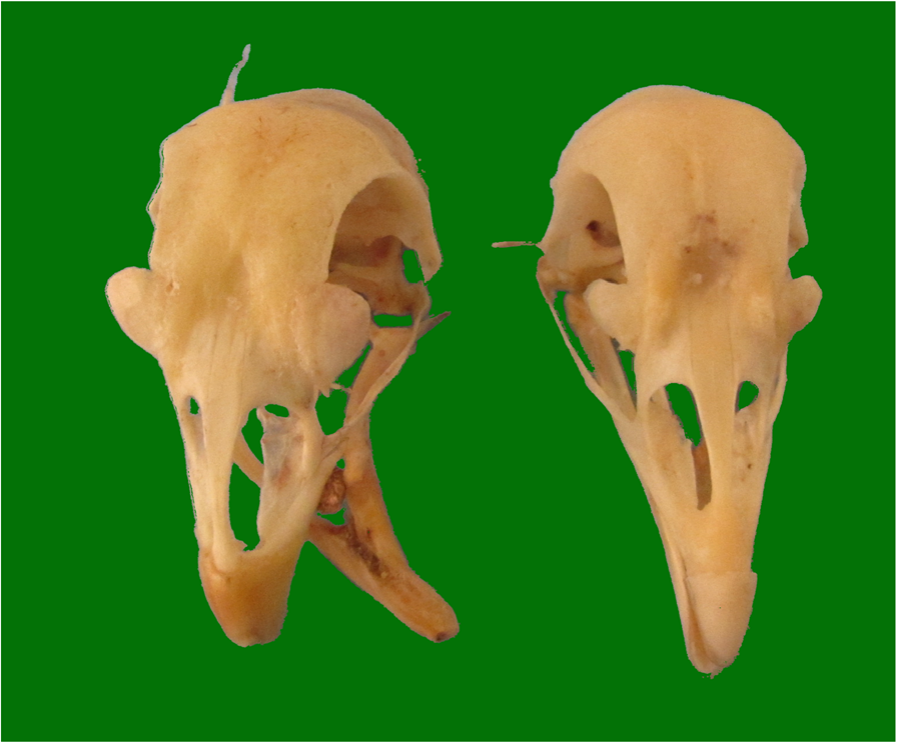
Skull anatomy of Beijing-You chickens with deformed and normal beaks. Compared with the normal beak, the lower mandible crossed left or right with an elongation of one of the rami

The beak is an external anatomical structure of birds, which consists of an upper and a lower mandible [1]. In a similar way to lips and teeth in mammals, the beak functions in birds primarily for feeding and drinking. It also has differentuses because of its diversity in shape between bird species [2–4]. For example, crossbill species like *Loxia* are characterized by the mandibles with crossed beaks [5], which is an adaptation enabling them to extract seeds from the cones they live on. However, for most bird species, especially for poultry, crossed beaks represent a malformation or an abnormality [6–9]. According to our previous investigations, a frequency of beak deformity (normally the lower mandible crossed left or right randomly) of 1 to 3% was found in several indigenous chickens, including Silkies, Huxu, and Beijing-You (BJY) chickens. Figures 1 and 2 show examples of the deformed and normal beaks of BJY chickens. Birds with crossed beaks can be clearly determined within 4 weeks after birth and cannot be rehabilitated later. Birds with deformed beaks have reduced feed intake, inhibited growth, poor production performance, and shorter survival. In the absence of known environmental factors contributing to the malformation, birds with a deformed beak are present consistently in each generation and cannot be eliminated simply on the basis of the observed phenotype. Furthermore, pedigree information indicated that the birds with a deformed beak can be traced back to limited number of ancestors. This suggested that genetic effects underlie this complex trait [10]. In addition, our previous mating experiment suggested that beak deformity could be a complex trait regulated by multiple genes. Several recognized genetic factors associated with beak shapes include genes such as *ALX1* [11], *HMGA2* [12], *FGF8* [13], *Shh* [14], *BMP4* [15, 16], and *CaM* [17]. The over-expression of *HOXA1* and *HOXD3* genes may result in beak deformity in chicks [18]. Previous digital gene expression (DGE) analysis in our laboratory identified several new genes (e.g. *LOC426217*) and pathways (e.g. unsaturated fatty acid biosynthesis and glycerolipid metabolism) [10, 19] that are involved in beak deformity.

With the advance of high-throughput genotyping platforms and computing technology, molecular markers and genes related to complex traits or diseases have been identified using genome-wide association studies (GWASs) [20–23]. This approach is powerful to identify a single SNP with a notable effect, especially for phenotypes that are determined by a unique gene or mutation. However, more complex traits are determined by genetic variants that may have significant combined effects instead of a single-SNP effect [24]. Pathway-based GWAS [25, 26] is one of the strategies and statistical approaches that considers multiple genetic variants as SNPs or genes in a biological pathway to understand the genetic contributors of complex traits [27, 28].

In the present study, with the aim of identifying the genetic backgrounds of beak deformity in chickens, 48 BJY birds with deformed beaks and 48 normal ones were genotyped using the 600 K high-density (HD) genotyping array [29]. ROADTRIPS [30] and SNP ratio test (SRT) [31] software were used to identify the associated SNPs and pathways, respectively. The combined analyses extend our understanding of the genetic basis of this trait.

## Results

### Single SNP-based association study

**Fig. 3:**
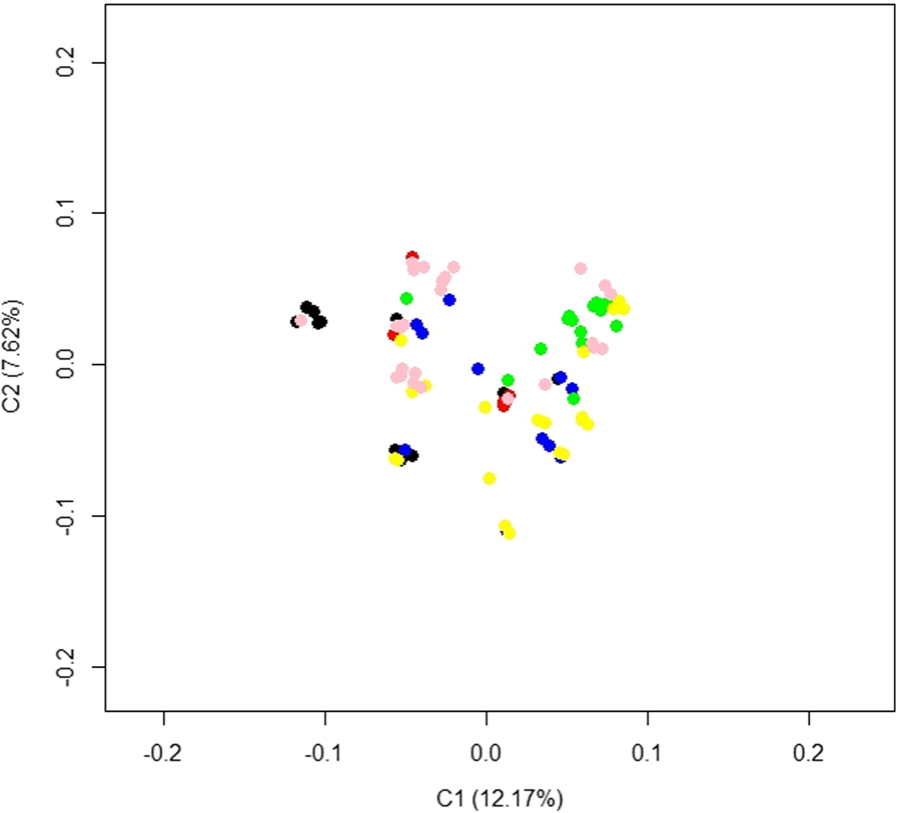
Population structure evaluated by the first two principal components. Six colors indicate the progeny of six males

In this study, a total of 48 birds with deformed beaks (case) and 48 normal birds (control) were genotyped using the Affymetrix Chicken 600 K HD genotyping arrays. According to the quality control (QC) criteria, one individual that had > 10% missing genotypes was removed. Additionally, 4505 SNPs with call rate < 90% and 120,261 SNPs with minor allele frequency (MAF) < 5% were excluded. As a result, 95 individuals and 429,539 SNPs were carried forward for subsequent analyses. The distribution of SNPs that passed QC in the chicken genome is presented in Table 1. A total of 21,984 independent SNP markers were obtained using multidimensional scaling (MDS) analysis using the first two principal components (Fig. 3), indicating no obvious population substructure in the birds.

**Table 1:** Distribution of SNPs that passed quality control on the chicken genome

Chromosome	No. SNPs	Physical length (Mb)^c^	Marker density (kb/SNP)	5% chromosome-wide significance threshold^d^
0^a^	5979	–	–	8.36E-06
1	81,335	195.3	2.40	6.15E-07
2	50,755	148.8	2.93	9.85E-07
3	45,028	110.4	2.45	1.11E-06
4	33,760	90.2	2.67	1.48E-06
5	24,678	59.6	2.41	2.03E-06
6	16,436	35.0	2.13	3.04E-06
7	17,826	36.2	2.03	2.80E-06
8	13,667	28.8	2.10	3.66E-06
9	14,291	23.4	1.64	3.50E-06
10	14,631	19.9	1.36	3.42E-06
11	10,749	19.4	1.80	4.65E-06
12	11,590	19.9	1.72	4.31E-06
13	8589	17.8	2.07	5.82E-06
14	10,062	15.2	1.51	4.97E-06
15	7697	12.7	1.64	6.50E-06
16	273	0.5	1.96	1.83E-04
17	7113	10.5	1.47	7.03E-06
18	7292	11.2	1.54	6.86E-06
19	6517	10.0	1.53	7.67E-06
20	7321	14.3	1.95	6.83E-06
21	6786	6.8	1.00	7.37E-06
22	2877	4.1	1.42	1.74E-05
23	4584	5.7	1.25	1.09E-05
24	5541	6.3	1.14	9.02E-06
25	1860	2.2	1.18	2.69E-05
26	4485	5.3	1.19	1.11E-05
27	3784	5.2	1.38	1.32E-05
28	3862	4.7	1.23	1.29E-05
LGE22C19W28_E50C23^b^	124	1.0	7.78	4.03E-04
LGE64^b^	47	0.8	17.02	1.06E-03
Total	429,539	921.2	2.46	1.16E-07

^a^These SNPs are not mapped to any chromosome

^b^Two linkage groups

^c^The physical length of the chromosome was based on the genome build Gallus_gallus-4.0/galGal4 (Nov. 2011)

^d^Bonferroni-corrected 5% chromosome-wise significance threshold = 0.05/number of SNPs after quality control

**Fig. 4:**
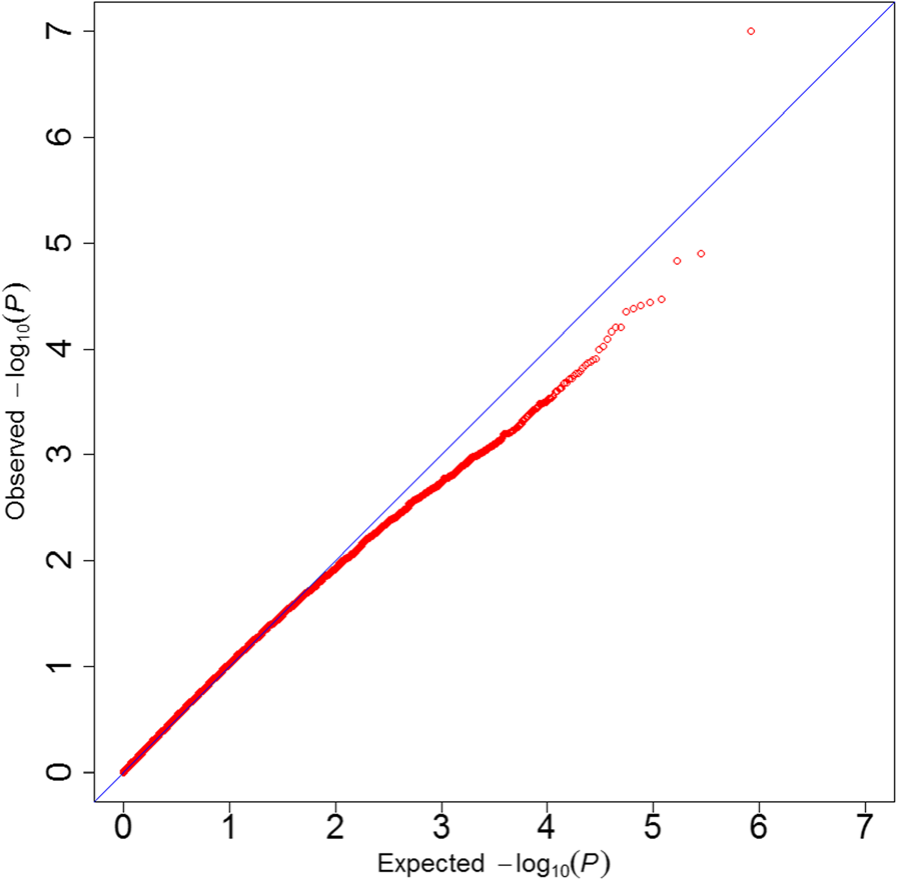
Quantile-quantile (Q-Q) plot of the genome-wide association analysis using ROADTRIPS. The x-axis shows the expected *P*-values under the null hypothesis and the y-axis shows the observed *P*-values. The value of inflation factor λ was 1.045

**Fig. 5:**
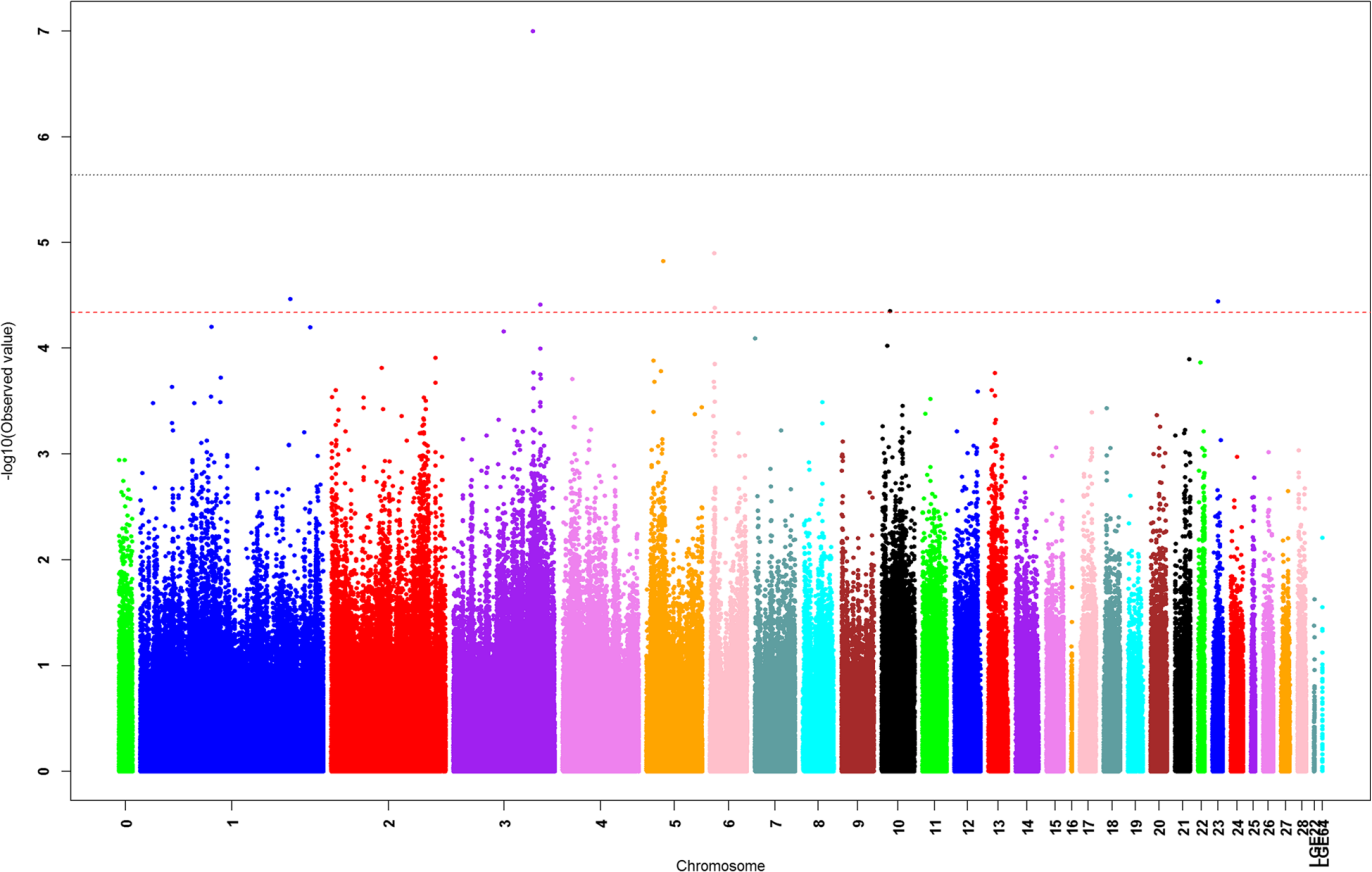
Manhattan plots showing association of all SNPs with the beak deformity trait using ROADTRIPS. SNPs are plotted on the x-axis according to their positions on each chromosome against their association with this trait on the y-axis (shown as -log_10_ (*P*-value)). The red dashed line indicates suggestive genome-wide significance (*P*-value = 4.55E-5), and the grey dashed line shows genome-wide 5% significance with a *P*-value threshold of 2.27E-6

ROADTRIPS was used for the traditional SNP-based association analysis. In total, one associated SNP (5% genome-wide significance (2.27E-6, 0.05/21,984)) on GGA 3 (chicken chromosome 3), and seven suggestively associated SNPs (4.55E-5, 1/21,984) on GGAs 1, 3, 5, 6, 10, and 23 were detected. The detailed information of the SNP reaching 5% genome-wide significance and the seven suggestively associated SNPs is shown in Tables 2 and 3, respectively. For a SNP-based GWAS, the genomic inflation can be determined and calculated by a lambda (λ) value. If the analysis results of the data follow the normal chi-squared distribution (no inflation), the expected λ value is 1. In this study, for the Quantile-quantile (Q-Q) plot presented as Fig. 4, the λ value was 1.045, which means a minimal and acceptable inflation at the upper tail of the distribution. Therefore, we believe the result was reliable. The global view of *P*-values (in terms of -log_10_ (*P*-value)) for all SNPs was represented by a Manhattan plot, as shown in Fig. 5. The raw results of all the SNPs are described in Additional file 1: Table S1.

**Table 2:** SNPs associated with beak deformity with 5% genome-wide significance

SNP ID	Chromosome	Physical position (bp)	Nearby^a^ genes	*P*-value
rs313625170	3	87,528,719	*LOC421892*, *TINAG*, *LOVL5*, *LRRC1*, *GCLC*, *KLHL31*, *LOC107053134*	9.97E-08

^a^Genes located within 5 Mb upstream or downstream of the significant SNPs

**Table 3:** SNPs suggested to be associated with beak deformity

SNP ID	Chromosome	Physical position (bp)	Nearby^a^ genes	*P*-value
rs316010119	6	4,029,716	*RET*, *MBL2*, *RASGEF1A*	1.25E-05
rs317906090	5	18,672,451	*LDLRAD3*, *COMMD9*, *SLC1A2*, *RAG1*	1.49E-05
rs313002111	1	161,794,494	*OLFM4*, *DIAPH3*, *TDRD3*	3.42E-05
rs313486014	23	2,844,607	*PTPRU*, *STMN1*	3.61E-05
rs16327528	3	95,937,095	*YWHAQ*, *TAF1B*	3.87E-05
rs317725514	6	4,455,292	*BMS1*, *RET*	4.16E-05
rs14944750	10	5,358,795	*MIR204–2*, *APBA2*, *ADAL*	4.42E-05

^a^Genes located within 5 Mb upstream or downstream of the suggestively associated SNPs

### Pathway-based association study

**Fig. 6:**
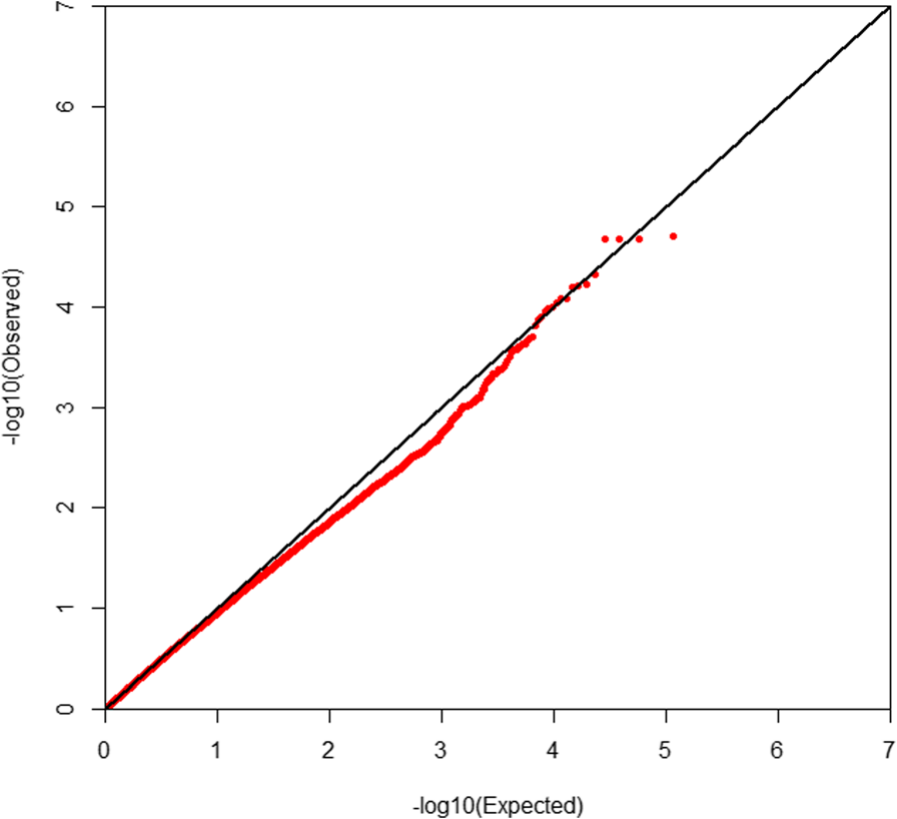
Quantile-quantile (Q-Q) plot for the SNPs used in the pathway-based GWAS. The x-axis shows the expected *P*-values under the null hypothesis and the y-axis shows the observed *P*-values. The value of inflation factor λ was 0.962

The same genotyping dataset was used in this analysis. Based on the QC and SNP selection criteria (see Methods), a total of 149 pathways were finally selected from chicken pathway dataset, covering 128,072 SNPs (with 115,222 SNPs and 12,850 SNPs involved in the unique and multiple pathways, respectively). After correction for genetic relationships, we conducted a standard association analysis in PLINK for both the original and 100 randomized phenotype datasets. The association tests for the original dataset resulted in about 5000 nominally significant SNPs (unadjusted *P* < 0.05, Additional file 2: Table S2). The Q-Q plot is shown in Fig. 6, and the λ value was 0.962.

**Fig. 7:**
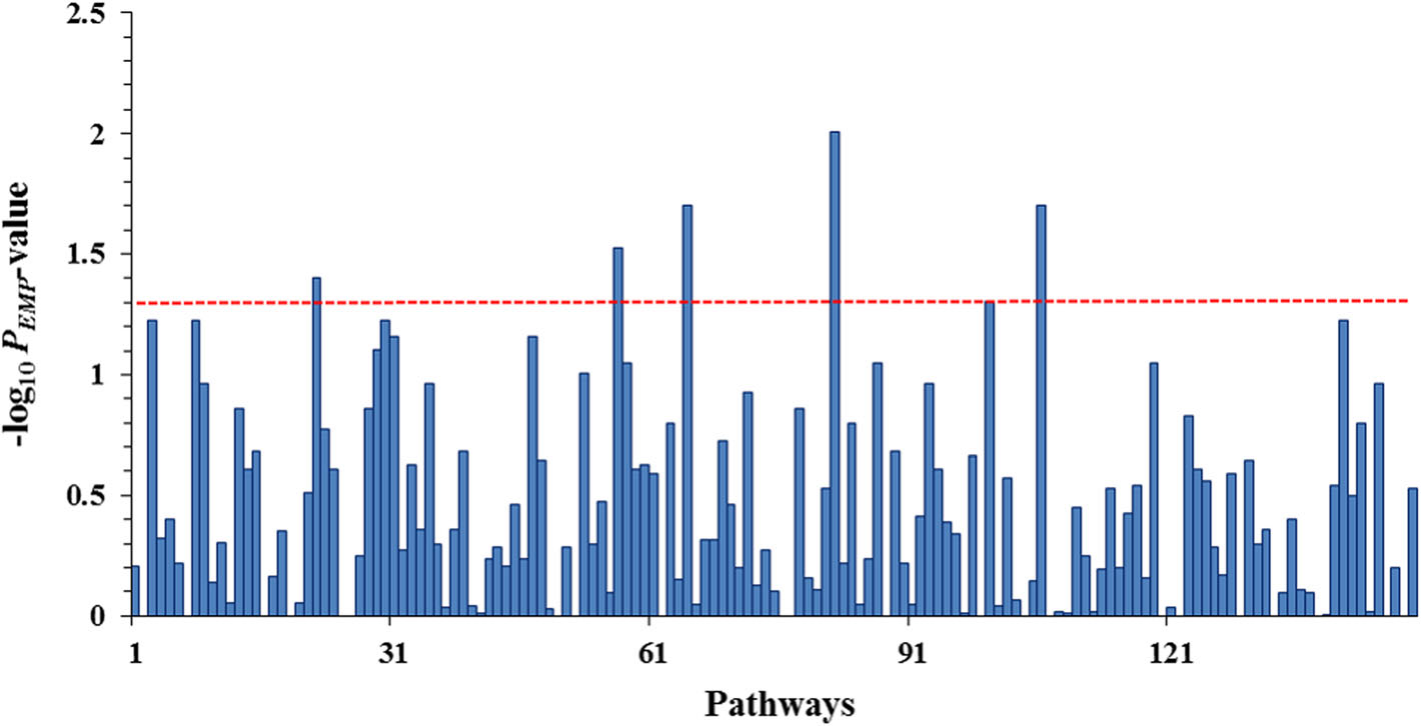
The -log_10_ (*P*_*EMP*_-value) values of the 149 pathways for the beak deformity trait

An SRT program was then applied to investigate the associations between beak deformity and the 149 pathways. As a result, we identified six associated pathways (*P*_*EMP*_ < 0.05), including the ribosome pathway, the oocyte meiosis pathway, the pantothenate and CoA biosynthesis pathway, the pyruvate metabolism pathway, the glycine, serine, and threonine metabolism pathway, and the calcium signaling pathway (Table 4 and Fig. 7). The detailed information of each pathway is provided in Additional file 3: Table S3.

**Table 4:** Six pathways significantly associated with beak deformity

Pathway ID	Definition	*P*_*EMP*_-value	Ratio
gga03010	Ribosome	9.90E-03	194/1431 (13.6%)
gga04114	Oocyte meiosis	1.98E-02	98/433 (22.6%)
gga00770	Pantothenate and CoA biosynthesis	1.98E-02	32/95 (33.7%)
gga00620	Pyruvate metabolism	2.97E-02	63/231 (27.3%)
gga00260	Glycine, serine and threonine metabolism	3.96E-02	71/366 (19.4%)
gga04020	Calcium signaling pathway	4.95E-02	519/4106 (12.6%)

Furthermore, we prepared plots of the number of significant SNPs, the number of total SNPs, the number of genes, the total length of genes, and the average length of genes for each pathway, against the -log_10_ *P*_*EMP*_-value of the selected pathways to investigate the effects of potential factors on SRT’s detection of the pathways. The associations between these factors and the significance of the pathways confirmed the utility of this strategy and permutations to reduce any bias caused by the SNPs, genes, gene sizes, and pathways (Additional file 4: Figure S1).

## Discussion

Excluding other environmental contributing factors, beak deformity is observed in each generation of BJY chickens and cannot be eliminated based solely on the phenotype. The heritability of beak deformity was estimated at 0.12 (SE = 0.17) by ASReml-R 3 (https://www.vsni.co.uk/software/asreml-r/) using the SNP data in this study. This indicated that this trait is genetically determined. However, previous studies did not validate the role of any candidate genes for beak shapes and beak length in beak malformation [32, 33]. Birds with deformed beaks have much higher mortality and lower fertility rates, making it difficult to obtain individuals for genetic analysis. Eventually, a small-sized population with only 48 birds with deformed beaks was collected and used in the present study. Baird et al. [34] used 96 samples to identify three main chromosomal regions related to cranial cruciate ligament rupture in Newfoundland dogs. Fels and Distl [35] identified one significant and four suggestively significant SNPs related to canine hip dysplasia in 96 German shepherd dogs. These previous studies indicated that GWAS is also effective to study complex traits and diseases with a small sample size. In the present study, based on the case-control design, the genotype data of 48 birds with deformed beaks and 48 normal chickens were used for the single SNP and pathway-based GWAS analyses.

In the single SNP-based study, ROADTRIPS was used to increase the power of the association study [30]. PLINK was also used in the same way, however, it only identified one suggestively associated SNP (Additional file 5: Figure S2), which was exactly the 5% genome-wide significant SNP (rs313625170) identified by ROADTRIPS. This SNP is located on GGA 3, 6.7 kb, 24 kb, 63 kb, and 0.13 Mb upstream of *LOC421892* (Transcription elongation factor B polypeptide 3-like,*TCEB3*-like), *KLHL31* (Kelch like family member 31), *GCLC* (Glutamate-cysteine ligase catalytic subunit), and *ELOVL5* (ELOVL fatty acid elongase 5) genes, respectively, and 26 kb and 0.21 Mb downstream of *LRRC1* (Leucine rich repeat containing 1) and *TINAG* (Tubulointerstitial nephritis antigen) genes, respectively. This SNP is also located in the intron of *LOC107053134*, which is a predicted gene. The seven suggestively associated SNPs, rs313002111, rs16327528, rs317906090, rs316010119, rs317725514, rs14944750, and rs313486014, are located on GGAs 1, 3, 5, 6, 6, 10, and 23, respectively. They are located within, downstream or upstream of genes including *TDRD3* (Tudor domain containing 3), *RET* (Ret proto-oncogene), and *STMN1* (Stathmin 1). Two genes identified here, *LOC421892* and *TDRD3*, were also highlighted in our former transcriptome study with a false discover rate (FDR) < 0.01 and |log_2_-Ratio (deformed/normal)| ≥ 1.5 [10], where *LOC421892* was down-regulated and *TDRD3* was up-regulated in the deformed beaks. The *LOC421892* gene is a homolog of *TCEB3* (also known as *ELOA*) gene, which is related to von Hippel-Lindan (VHL) [36] and nonsyndromic cleft lip and palate (NSCLP) [37] in human. It is the gene nearest to the significant SNP (rs313625170) identified here and could be the first candidate gene contribute to the formation of a deformed beak. *TDRD3*, located 0.19 Mb downstream of a suggestively significant SNP (rs313002111), is related to chromatin binding and methylated histone binding [38]. Mutation of *TDRD3* is associated with human Fragile-X syndrome [39] and primary ovarian insufficiency [40]. *RET*, located 0.26 Mb upstream and 92 kb downstream of two suggestively significant SNPs, rs316010119 and rs317725514, respectively, might also play an important role in the beak shaping. Its gene product is involved in neural crest development [41]. *RET* can undergo oncogenic activation in vivo and in vitro by cytogenetic rearrangement [42]. As reported by Schneider and Helms [43], the origin and evolution of the beak in birds is strongly associated with neural crest cells. Moreover, mutations in *RET* are also associated with the disorders such as multiple endocrine neoplasia, type IIA, multiple endocrine neoplasia, type IIB, Hirschsprung disease, and medullary thyroid carcinoma [44]. In addition, its interacting gene, *LRIG2*, was also identified as one of the most promising candidate genes underlying this trait in our previous CNV study within the same genotyped data used here [45]. Thus, the *RET* and *LRIG2* genes might jointly contribute to the development of deformed beaks. Similarly, *STMN1*, also named Oncoprotein 18, located 0.35 Mb downstream of another suggestively significant SNP (rs313486014), is involved in myelodysplastic syndromes [46], and is related to chick retina [47] and nasopharyngeal carcinoma [48]. These four genes may be related to beak deformity in chickens according to their positions, known functions, and our former studies.

In contrast to the single-SNP GWAS, pathway-based GWAS can identify the biological pathways that are useful to interpret the genetic basis of complex traits. The limits of pathway annotation and the power of the GWAS dataset, mean that adequate SNP coverage is essential for a pathway to be effectively tested [49]. This method has two primary advantages [31]: (1) it avoids issues arising from linkage disequilibrium (LD) by using the same SNPs in all simulations. Only pathways with additional significant SNPs, not merely arising from LD, are deemed as significant; (2) it uses individual level data in its simulations, which maximizes the information available to test pathway hypotheses. Furthermore, we also conducted some plots to confirm that the SNP number, gene number, and gene length in each pathway did not influence the results of SRT.

Six associated pathways were obtained, of which, the calcium signaling pathway (Additional file 6: Figure S3) has the most potential to be involved in beak deformity. Calcium ions are important for cellular signaling, because once they enter the cytoplasm they exert allosteric regulatory effects on many enzymes and proteins [50]. Calcium can act in signal transduction resulting from activation of ion channels or as a second messenger caused by indirect signal transduction pathways, such as those of G protein-coupled receptors [51]. In neurons, concomitant increases in cytosolic and mitochondrial calcium are important for the synchronization of neuronal electrical activity with mitochondrial energy metabolism. Thus, calcium plays an important role in regulating various neuronal processes [52]. Several studies have shown that calcium was highly associated with beak shape in birds. A previous study demonstrated differential expression of calmodulin between finches with different beak types [17]. The structure of a toucan beak was found to be a sandwich composite with an exterior of keratin and a fibrous network of closed cells comprising calcium-rich proteins [53]. Lamichhaney [11] found that among the 15 most significant genomic regions related to beak shape, six harbored genes associated with craniofacial and/or beak development in mammals or birds were identified, including calmodulin. From the previous studies in our laboratory, Zhu et al. [54] revealed that the calcium content of the beak was 7.5–12%. Liu et al. [55] observed that the over-expression of parvalbumin, a calcium ion-binding protein, could result in beak deformity in chickens, as assessed using iTRAQ-based proteomic analysis. Taken together, these results suggest that the calcium signaling pathway could be a key factor related to the beak deformity trait in chickens.

## Conclusions

In conclusion, one 5% genome-wide significant SNP and seven suggestively significant SNPs that may be involved in the beak deformity trait were identified, using the single SNP GWAS. Four candidate genes, *LOC421892*, *TDRD3*, *RET*, and *STMN1*, were identified as the most promising genes underlying this trait. Simultaneously, six pathways were found to be associated with this trait using the pathway-based GWAS, where the calcium signaling pathway may be the most important. Overall, our findings are worthy of further functional characterization to reveal the pinpoint causes of beak deformity and the underlying the mechanism of this disorder in chickens.

## Methods

### Animals and DNA samples collection

Six males and 12 females with deformed beaks from a BJY chicken population, a local breed conserved by Institute of Animal Science, Chinese Academy of Agricultural Sciences (IAS, CAAS, Beijing, China), were used for a random mating experiment to produce 921 offspring. The incidence of beak deformity birds was 7.82%, which was higher than that observed previously in the normal population (up to 3%). Forty-eight birds with deformed beaks and 48 normal birds were randomly selected from the offspring at 20 days of age, when the crossed beaks could be clearly identified, and used for genotyping. All of these birds were observed until 90 days of age before blood collection to make sure that the phenotype was stable. Blood samples were collected from the brachial vein by venipuncture. Genomic DNA (gDNA) was isolated from blood samples using the phenol-chloroform method. The purity and concentration of the gDNA samples were measured using a Nanodrop ND-2000 spectrophotometer (Thermo Scientific, MA, USA). The final concentration was adjusted to 50 ng/μL. gDNA samples with an A260/280 ratio of 1.8–2.0 were submitted for genotyping.

### Genotyping and quality control

gDNA samples were genotyped using 600 K Affymetrix Axiom HD genotyping arrays containing 580,954 SNPs based on the Affymetrix GeneChip platform [29]. The genotypes were identified using Affymetrix Genotyping Console (Version 4.2, Affymetrix Inc., Santa Clara, CA, USA) and a custom cluster file developed from the 96 (48 deformed and 48 normal) samples. Following the case-control design, stringent QC procedures were performed for the genotype data using PLINK [56] (Version 1.07, http://zzz.bwh.harvard.edu/plink/). First, individuals with > 10% missing genotypes were excluded (*n* = 1). Second, out of the initial full set of 554,305 effective SNPs in this study, we discarded those with a call rate < 90% (*n* = 4505) and those having a minor allele frequency (MAF) < 0.05 in all birds (*n* = 120,261). The Hardy-Weinberg equilibrium (HWE) was not used to filter data in view of the small population.

### Population stratification assessment

To evaluate the existence of a population substructure among the individuals, the classical MDS was performed in PLINK using the following procedures: (a) autosomal SNPs were subjected to LD-based pruning to ensure that uncorrected LD did not distort the analysis. Thus, the remaining SNPs within a window size of 50 SNPs and a step of 10 SNPs had pairwise r^2^ < 0.2; (b) the pairwise identical-by-state (IBS) distance among the 95 individuals was calculated using the remaining 21,984 SNPs; (c) the first two MDS dimensions were extracted via the “MDS-plot” command and visualized in R (version 3.2.0, www.r-project.org).

### Single SNP-based association analysis

The *R*_*M*_ test was performed for the association analysis in ROADTRIPS (Version 1.2) [30]. An important advantage of ROADTRIPS is that it can deal with data with a known pedigree structure as well as population admixture in an association test by constructing an empirical covariance matrix from genome-wide SNP data to adjust for potential population admixture and for genetic connectedness among individuals in both the control and case groups. The *P*-value was corrected using a strict Bonferroni adjustment based on LD pruning [57]. The sum of the independent LD blocks plus singleton markers were used to define the number of independent statistical comparisons [58]. Finally, 21,984 independent tests were used to determine the *P*-value thresholds, including 5% genome-wide significance (2.27E-6, 0.05/21,984) and suggestive association (4.55E-5, 1/21,984). The Q-Q plot and the Manhattan plot of GWAS for this trait were produced using the “gap” package [59] in R.

### Pathway-based association analysis

We retrieved all the pathways in chicken (Additional file 7: Table S4) from the KEGG [60] pathway database (http://www.genome.jp/kegg/) to identify pathways that potentially contribute to the beak deformity trait in the chicken. A total of 162 annotated pathways were collected for analysis. Only SNPs located within or 50 kb upstream or downstream of a gene were selected to create a file linking pathways and SNP information (Additional file 8: Table S5). In addition, if a SNP was involved in multiple pathways, the SNP and the pathways were both included in the analysis. A SRT program (Version 3, https://sourceforge.net/projects/snpratiotest/) was employed for the analysis. The SRT compared the proportion of the significant SNPs (unadjusted *P* < 0.05 in the single SNP analysis) to all the SNPs that are part of a pathway and computed an empirical *P*-value (*P*_*EMP*_) based on comparisons to ratios in simulated datasets where the assignment of case/control status had been randomized. The simulated datasets were constructed from the original dataset, preserving the original case/control ratio, but randomizing the assignment of case/control status among individuals. The SRT accepts files in the PLINK binary format and allows the user to prepare randomized phenotype datasets. The *P*_*EMP*_ for a particular pathway, = (*s* + 1)/(*N* + 1), where *s* is the number of simulated datasets that produce a ratio greater than or equal to the original ratio and *N* is the number of permutations [61]. The pipeline of SRT is shown in Additional file 9: Figure S4.

## Additional files

Additional file 1: **Figure S1.** Significance of the pathway (−log10 (*P*_*EMP*_-value)) versus: (a) the number of significant SNPs in the pathways, (b) the number of SNPs in the pathways, (c) the number of genes in the pathways, (d) total length (kb) of genes in the pathways, and (e) average length (kb) of the genes in the pathways. The *P* = 0.05 cut-off is highlighted by a vertical red line. (JPG 105 kb)

Additional file 2: **Figure S2.** Manhattan plots showing the association of all SNPs with beak deformity trait using PLINK. SNPs are plotted on the x-axis according to their positions on each chromosome against their association with this trait on the y-axis (shown as -log_10_ (*P*-value)). The red dashed line indicates suggestive genome-wide significance (*P*-value = 4.55E-5). (TIF 2715 kb)

Additional file 3: **Figure S3.** The calcium signaling pathway. (TIF 27 kb)

Additional file 4: **Figure S4.** The pipeline of SRT (Referred to the SRT manual). (TIF 100 kb)

Additional file 5: **Table S1.** Raw results of the single SNP-based association study. (XLSX 21782 kb)

Additional file 6: **Table S2.** Single SNP-based association analysis of the selected SNPs used in the pathway-based association study. (XLSX 5391 kb)

Additional file 7: **Table S3.** The detailed information of each pathway based on the pathway-based association analysis. (XLSX 15 kb)

Additional file 8: **Table S4.** The detailed lists of all the chicken pathways used in the pathway-based association analysis. (XLSX 14 kb)

Additional file 9: **Table S5.** The detailed lists of pathways and SNPs information used in the pathway-based association analysis. (XLSX 3841 kb)

## Abbreviations

*ALX1*: ALX homeobox 1
BJY: Beijing-You
*BMP4*: Bone morphogenetic protein 4
*CaM*: Calmodulin
CNV: Copy number variation
DGE: Digital gene expression
*ELOA*: Elongin A
*ELOVL5*: ELOVL fatty acid elongase 5
*FGF8*: Fibroblast growth factor 8
*GCLC*: Glutamate-cysteine ligase catalytic subunit
gDNA: Genomic DNA
GWAS: Genome-wide association study
HD: High-density
*HMGA2*: High mobility AT-hook 2
*HOXA1*: Homeobox A1
*HOXD3*: Homeobox D3
HWE: Hardy-Weinberg equilibrium
IBS: Identical-by-state
iTRAQ: Isobaric tags for relative and absolute quantification
*KLHL31*: Kelch like family member 31
LD: Linkage disequilibrium
*LRRC1*: Leucine rich repeat containing 1
MAF: Minor allele frequency
MDS: Multidimensional scaling
*P*_*EMP*_: Empirical *P*-value
QC: Quality control
Q-Q: Quantile-quantile
*RET*: Ret proto-oncogene
*Shh*: Sonic hedgehog
SNPs: Single nucleotide polymorphisms
SRT: SNP ratio test
*STMN1*: Stathmin 1
*TDRD3*: Tudor domain containing 3
*TINAG*: Tubulointerstitial nephritis antigen
