# Supplementary figures and images for: Single SNP- and pathway-based genome-wide association studies for beak deformity in chickens using high-density 600K SNP arrays
Source: BMC Genomics. 2018 Jun 28;19:501. doi: 10.1186/s12864-018-4882-8 (PMC6022433; doi:10.1186/s12864-018-4882-8)

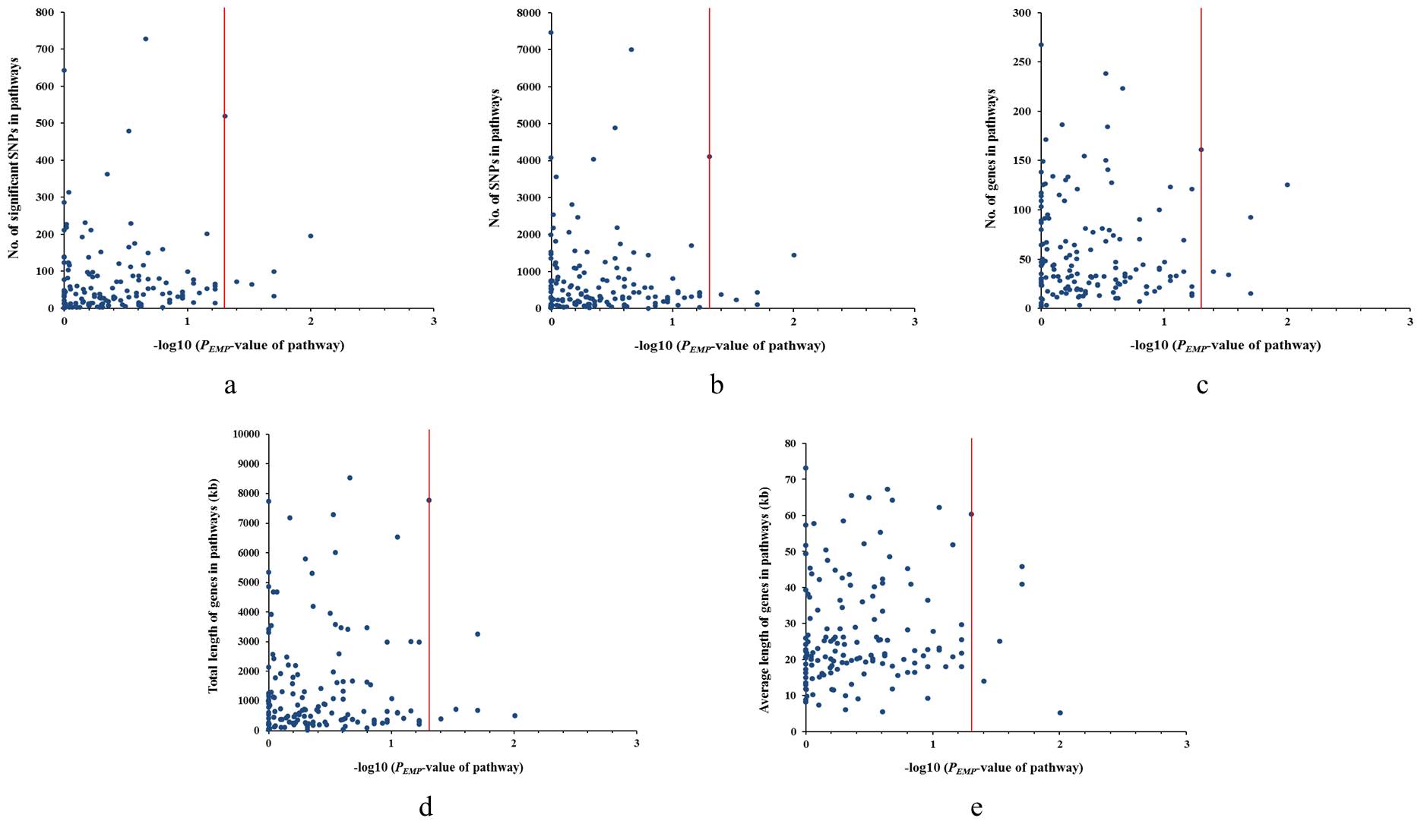

Supplement: Supplementary file 1 — Figure S1. Significance of the pathway (−log10 (PEMP-value)) versus: (a) the number of significant SNPs in the pathways, (b) the number of SNPs in the pathways, (c) the number of genes in the pathways, (d) total length (kb) of genes in the pathways, and (e) average length (kb) of the genes in the pathways. The P = 0.05 cut-off is highlighted by a vertical red line. (JPG 105 kb) [file 12864_2018_4882_MOESM1_ESM.jpg]

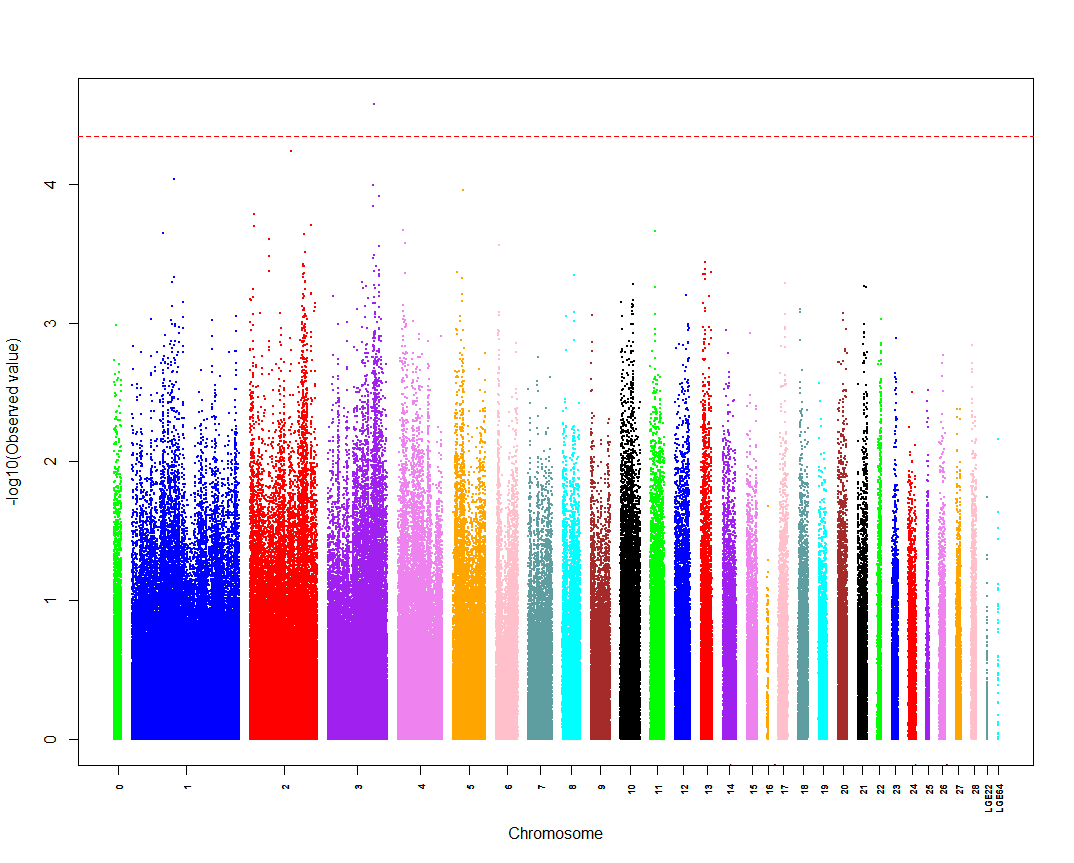

Supplement: Supplementary file 2 — Figure S2. Manhattan plots showing the association of all SNPs with beak deformity trait using PLINK. SNPs are plotted on the x-axis according to their positions on each chromosome against their association with this trait on the y-axis (shown as -log10 (P-value)). The red dashed line indicates suggestive genome-wide significance (P-value = 4.55E-5). (TIF 2715 kb) [file 12864_2018_4882_MOESM2_ESM.tif]

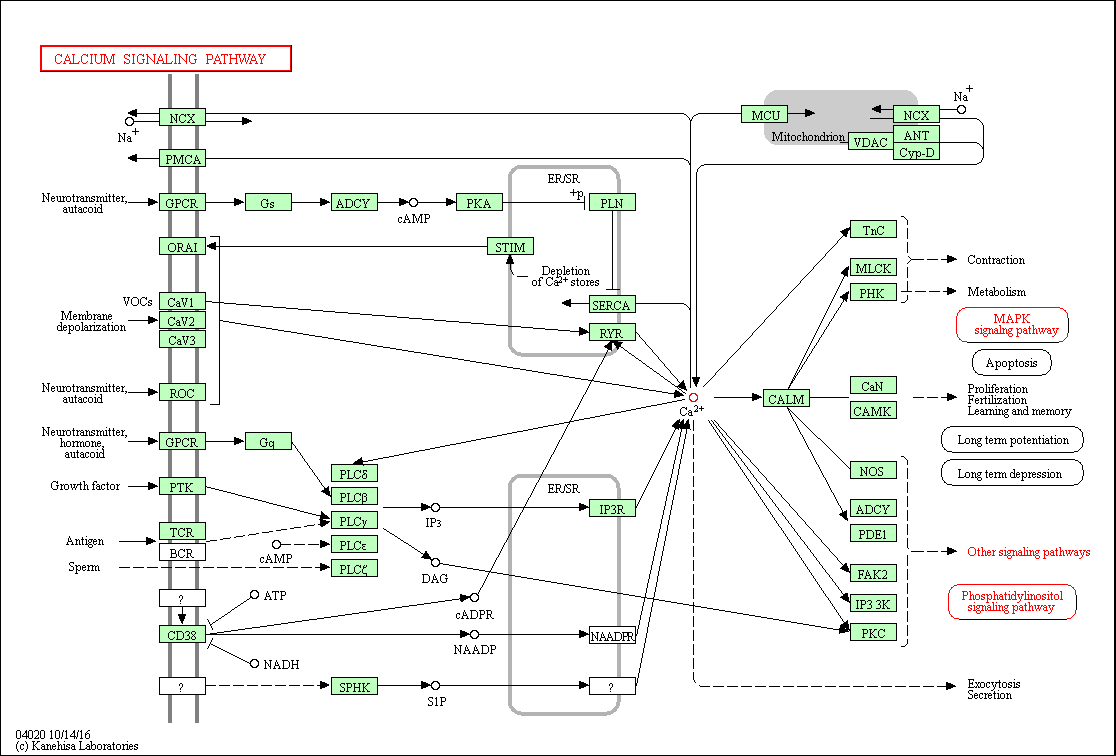

Supplement: Supplementary file 3 — Figure S3. The calcium signaling pathway. (TIF 27 kb) [file 12864_2018_4882_MOESM3_ESM.tif]

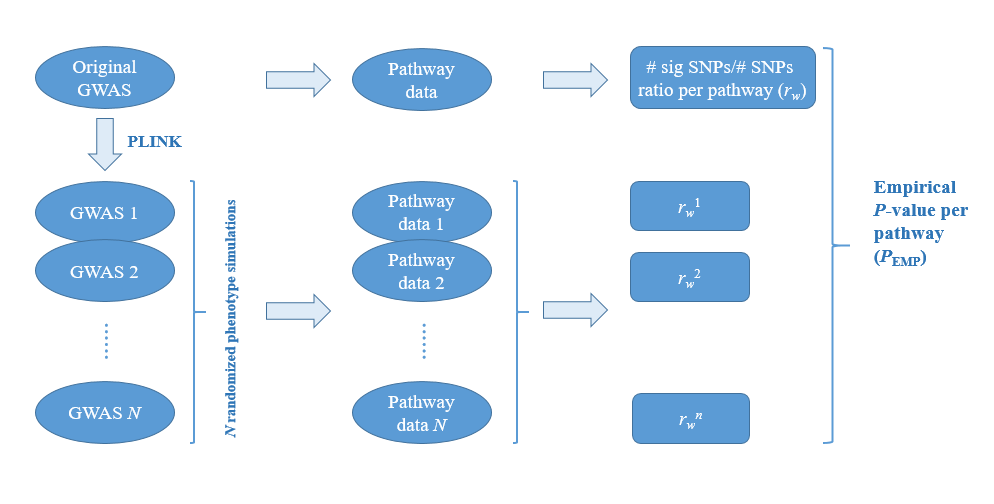

Supplement: Supplementary file 4 — Figure S4. The pipeline of SRT (Referred to the SRT manual). (TIF 100 kb) [file 12864_2018_4882_MOESM4_ESM.tif]
